# P-1897. Quality Improvement Initiative: Quantifying and Reducing Anti-infective Pharmaceutical Waste at a Tertiary Care Pediatric Hospital

**DOI:** 10.1093/ofid/ofae631.2058

**Published:** 2025-01-29

**Authors:** Shreya M Doshi, Esther Esadah, Johnny Yoko-Uzomah, Erica Howard, Craig A Shapiro, Aimee Dassner

**Affiliations:** Children's National Health System, Washington DC, District of Columbia; Children's National Hospital, Washington, District of Columbia; Childrens National Hospital, Washington, District of Columbia; Children's National Hospital, Washington, District of Columbia; Children's National Hospital, Washington, District of Columbia; Children's National Health System, Washington DC, District of Columbia

## Abstract

**Background:**

Pediatric hospitals have reported thousands of doses and dollars’ worth of waste from anti-infective drugs (anti-microbial, anti-fungal, anti-viral) every year. This has an environmental impact due to pharmaceutical waste incineration resulting in greenhouse gas emissions and contributes to drug shortages. Anti-infective drugs are prepared in advance for routine administration. When an order is modified or cancelled it cannot be used for another patient and gets discarded. The goal of this study was to calculate the anti-infective waste at our tertiary care pediatric hospital, to understand the key drivers and design education and QI interventions to reduce wasted doses.

**Figure 1:**
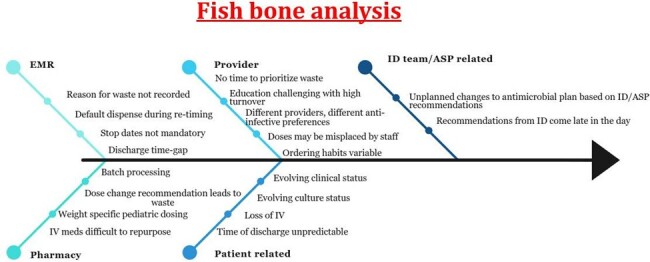
Fish bone diagram highlighting the reasons for waste

**Methods:**

Pharmacy data for waste reviewed (06/2022-03/2024). The reasons for wasted doses determined by point prevalence review of 75 charts on a single hospital day. PDSA 1 included creation of additional batch processing timings. PDSA 2 included education of providers on teams with high amounts of wasted orders held in 02/2024. Pharmacy leaders reminded teams daily on rounds about stop dates.

**Figure 2:**
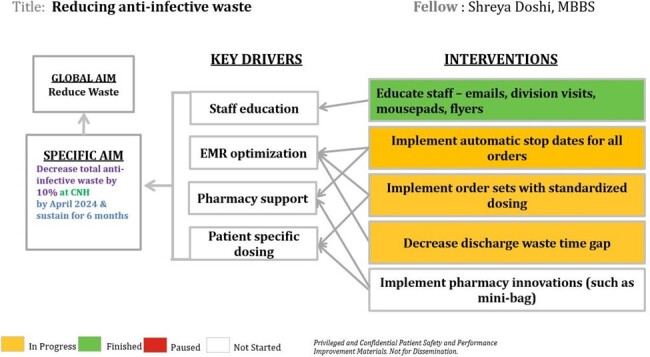
Key driver diagram

**Results:**

Total waste - 79,525 doses at a cost of $407,072 were wasted due to order changes in 2023. Top wasted anti-infectives include ampicillin-sulbactam, cefepime, cefazolin, ceftriaxone, vancomycin, and piperacillin-tazobactam.

PDSA 1 (Addition of batches for batch processing timing) & PDSA 2 (Provider education and awareness) showed significant reduction in waste per 1,000 doses as highlighted in figure 4.

**Figure 3:**
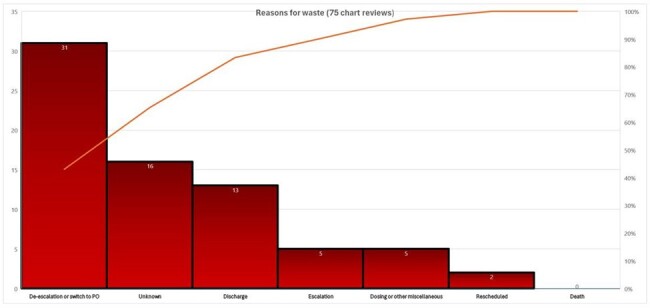
Reasons for waste on chart review of 75 patients

**Conclusion:**

Education around medication waste was created through this QI Initiative and was well received. Using the data collected, IT and pharmacy workflow interventions are being developed and will be evaluated including standardized dosing, 48 hour hard-stops for sepsis rule outs.A key intervention we are currently working on (PDSA 3) is to reduce the time gap between discharge of the patient and when the orders are discontinued on the EMR (identified during chart review). For our hospital this time gap was found to be 6.5 hours which results in large amounts of waste. Calculating environmental impact & Green House Gas emissions from the pharmaceutical waste (by measuring weight in kg) is another impact assessment metric we are working on.

**Figure 4:**
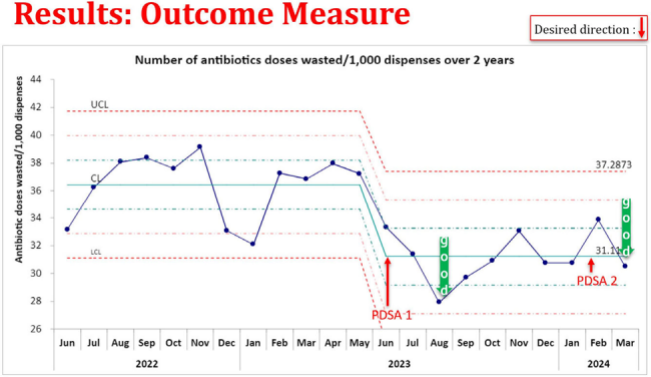
Run-chart of baseline waste data with the two PDSA cycles A decrease in the center line was observed following PDSA 1 that was sustained for greater than 6 months

**Disclosures:**

All Authors: No reported disclosures

